# Incomplete Coverage of Candidate Genes: A Poorly Considered Bias

**DOI:** 10.2174/138920207783591681

**Published:** 2007-11

**Authors:** Antonio Drago, Diana De Ronchi, Alessandro Serretti

**Affiliations:** Institute of Psychiatry, University of Bologna, Italy

**Keywords:** Candidate genes, association studies, psychiatry genetics, methodology.

## Abstract

Current genetic investigations are performed both on the basis of a rational and biologically based choice of candidate genes and through genome wide scans. Nonetheless, lack of replication is a common problem in psychiatric genetics as well as in other genetic fields. There are a number of reasons for this inconsistency, among them a well known but poorly considered issue is gene coverage. The aim of the present paper is to focus on this well known and defectively deemed bias, especially when a candidate gene approach is chosen. The rational and the technical feasibility of this proposal are discussed as well as a survey of current investigations. The known consistent methodology to fix this bias is also discussed.

## INTRODUCTION

By identifying heritable risk factors, genetic association studies will reasonably help to clarify the biological basis of psychiatric disorders, to identify the most consistent prognostic factors, to simplify the therapeutic choices in every day clinic management. Anyway, despite a worldwide impressive scientific effort, there are not many conclusive results so far. This lack of consistency may be due to some methodological bias. There are roughly two main ways to investigate the associations between genetic variants and phenotypes or endophenotypes: genome wide analysis and candidate gene analysis. Going genome wide is probably the way of the future, provided that the haplotype strategy turns out to be really applicable and effective across the genome [1]: however, genome wide investigations focused on psychiatric disorders reported no definitive association results so far [2]. On the other hand, the candidate gene approach showed some good results and some genetic variations proved to be highly informative: for example, the insertion/deletion polymorphism in the promoter of the serotonin transporter has been independently demonstrated to be associated with some aspects of psychiatric disorders [3-5], and some clinical guidelines for the use of pharmacogenetic testing for CYP4502D6 and 2C19 mutations are available already [6]. The genome wide and candidate methods are not thought to be competitive: inductive and deductive information can be drawn with mutual advantage. Anyway, a large part of genetic research, both with genome wide and candidate gene methodologies, leaded to poorly replicated results. This might be due to a list of reasons, and some of them are methodological by nature: biases related to phenotype definitions (diagnosis, symptom clusters), sample size, selection bias, treatment lack of homogeneity, statistical biases and the little impact of a single gene over clinical phenotypes [7, 8]. Beyond this well documented complexity, one well known but poorly considered bias is the incomplete coverage of genetic variations running within the gene which is under investigation. The aim of this paper is to consider if recent literature under estimated this last point. The reader will find a list of web resources helping to fix this bias at the end of the paper.

## THE ROLE OF SINGLE NUCLEOTIDE POLYMORPHISM (SNPs)

Since the finding of the first physical map of the human genome in 1956, the suggestion of random DNA markers to build a sequence map in 1980 [9], the completion of the sequence of human genome in 2003 and the first study with a genome wide technique (more than 44 clinical trials published in the last two years), the research community has now the possibility to do both wide (entire genome) and hypothesis related (single nucleotide polymorphism) genetic association studies. Briefly, DNA variations can occur at different levels: duplications, insertions, deletions and transpositions. The most frequent changes involve single nucleotide substitutions, insertions and deletions. There are two basic classes of polymorphisms: SNP (single nucleotide polymorphism) and VNTR (variable number of tandem repeats). A list of SNPs is available from public databases (http://www.hapmap.org/downloads/index.html.en), and a part of these variations has a relevant prevalence within the general population: they are considered as “common” above the 5% frequency [10]. There is a variable number of SNPs for each gene: the longer the gene, the higher the number of running variations within its sequence. It is not easy to define the average number of one gene mutations, but it may be reasonably said that one single mutation is not representative of a gene’s complete sequence. In fact, there might be other mutations within the same gene counteracting or enhancing the effect of the first one. We hypothesized that this simple, self – established statement is poorly considered by recent literature. To test this hypothesis, we surveyed the literature based on genetic association studies, inclusion criteria is listed below. A special attention might be paid to one of them: a sample size threshold (about one hundred patients) was chosen as inclusion criteria. This was meant to be closer to rational concerns about the studies’ economic burden than to statistics: budget represents a relevant part in every study design, and a cut off of one hundred patients may identify studies which could afford a complete genetic analysis. Some words should be spent about this controversial topic. The definition of a statistically correct sample size for an association study appears to be quite a complex task: small sample sizes are not representative, while bigger sample sizes are associated with the risk of higher false positive rates, as recently demonstrated by Sullivan [11]: his *in silico* investigation showed dramatic rates of false positive results with a sample of 500 cases and 500 controls, examined for a set of 10 SNPs. Conflicting with this, genome wide analysis performed on thousands of patients only reported mild significant results: the recent genome wide investigation by Fanous and colleagues [12] (n=1383), for example, shed some light on the boundaries between schizophrenia and schizotypy, but the level of significance (p=0.04 and p=0.02) does not allow to discard the possibility of a first type error occurrence. Similar considerations might then be done for other interesting recent findings: in an relevant paper by Bulayeva and colleagues, the genome wide investigation of a set of genetic isolated schizophrenic pedigrees revealed different pattern of association with psychiatric phenotypes between younger and older pedigrees [13]. The study of genetic isolated pedigrees lowered the occurrence of population stratification factors, and the wide investigated sample sizes (hundreds) were a relevant point of the study, but the level of significance that was reported (p < 0.05) may not be considered sufficiently protective. Furthermore, it must be noticed that, even with a higher level of significance (p<0.002) at the genome-wide analysis, the subsequent SNP investigation does not necessarily confirm the results [14]. Many factors probably influenced the statistical power of these studies and therefore, they all should be considered for statistically determined correct sample size: the haplotype Linkage Disequilibrium (LD) which varies along chromosomes and within genes, the assumed genetic path associated with disorders (additive or multiplicative), the prevalence of the investigated disorder in the general population, the prevalence of the alleles associated with the disorder, and the definition of the relative risk for the disorder may represent some examples of these interactions as far as they are all influent toward the definition of a sample size tailored to a fixed statistical detection power [15].

So, even in the simplest case, when a candidate gene approach is chosen, the index sample size will vary according to the disorder, to the haplotype Linkage Disequilibrium average value in the candidate gene, to the associated expected Odds Ratio and so on. Thus, the correct identification of the exact average sample size for a rational analysis appears to be quite a complex task, and it is anyway beyond the aim of this paper. If the reader is interested in this topic, De La Vega recently proposed an online free software meant to deal with these issues [15], and the recently published HAPMAP project phase II focuses on these topics in deep too [10], reader’s attention is then readdressed to these relevant publications. More simply, we choose to use the sample size to select appropriate studies for our survey on the basis that, given the economical effort leading to the selection of about two hundred persons (cases and controls), it would be worth performing a completer SNP selection, avoiding the simple bias of an incomplete investigation, which otherwise would detract from the scientific and economical effort of the study. According to this advice, we listed some recent association studies published in the last three years (July 2004 – July 2007) in the field of psychiatric genetic research, and pointed out the investigated/known genetic variations ratio for each gene in the studies. Pubmed database was used with the following criteria:

1. Key words: gene, polymorphism, schizophrenic, bipolar, depressive, anxiety, obsessive, panic, PTSD, phobic.
2. Sample size = 90-100
3. Limits: Humans, Clinical Trial, Meta-Analysis, English.

Results are presented in Table **1**, SNP data are collected from NCBI database (http://www.ncbi.nlm.nih.gov/).

Table **1** reports the low investigated/known genetic variation ratio in almost all considered studies: the complete SNP coverage of investigated gene then represents quite a new topic in nowadays literature [16]. There are good and well known reasons to revalue this point: first of all, it is rational to assume that every SNP occurring in the genetic coding sequence is crucial as far as it can be associated with a change in the secondary mRNA structure: that is why silent substitutions cannot be considered *a priori* as devoid of interest if they run in exons. Moreover, SNPs within the coding sequence can be associated with different aminoacid sequence, leading to secondary, tertiary or quaternary possibly different protein structures, and, as a consequence or independently from it, altered function. Not all the DNA sequence is expressed in every cell [17], but a part of it being silent or playing a regulatory role, probably influencing the differentiation and specialisation processes [18-20]: it might be expected then that a number of SNPs will occur in non coding DNA, and may be in - or close to - a promoter, enhancer, silencer or other regulating sequence. Promoters are usually close to exons sequence (TATA box usually locate at -25bp; CAAT box at -80bp; CG box up to -950bp), but enhancers and silencers can be located at a considerable distance from the coding stream or inside introns. Other reasons to consider intronic polymorphisms relevant to genetic investigations, arise from the DNA accessibility to RNA polymerases: it is commonly accepted that this mechanism is at least partially limited by methylation and acetylation [21-26], and since cytosine has been proposed to be a site of methylation, its variation due to a change in the genetic sequence could have an influence on the gene expression. Consistently, there is evidence that the T102C silent variation influence HTR2A gene expression [27] probably through this mechanism. Finally, an intron variation could be completely silent but in strong Linkage Disequilibrium with a not yet known variation, possibly involving genes or sequences still far to be hypothesized as relevant to the topic of the study.

As Table **2** shows, nowadays investigations of non exon variations are limited to sequences located 1 kbp around exons. For the above mentioned considerations, scans should be wider. To complete Table **2** we used temporal criteria (the last two years), and the following key words: intron, intronic, depressive, psychotic, schizophrenic, bipolar, personality, suicide, temperament, eating, anxiety, panic, obsessive, polymorphism, SNP. Pubmed served as database. As regard to SNPs, only studies with possible identification of genetic location are included. One study for each investigated variation is included.

Finally, 3’ and 5’ endings are also important regulatory regions, and it is reasonable to assume that modifications running in these genetic sequences, or sufficiently close to, might play a relevant role. 

Some lines of evidence encourage a complete analysis of single genes’ variations: Myer and colleagues recently found no significant difference in the promoter activity of HTR2A gene between the A- and G- allele of the -1438 locus when expressed with the major alleles at –1420 C/T and –783 A/G loci [28]. This was not consistent with some previous literature findings [29]; but when the minor allele G at -783 was found to be expressed with G-allele at -1438, the promoter activity was found to be significantly decreased. Consistently, a triallelic variant of the well known serotonin promoter polymorphism has been recently reported [30], and only the A allele carriers at the A/G SNP within 5-HTTLPR insertion polymorphism yield high mRNA levels, and the L(G) carriers actually behave like the low expressing short allele. This finding can explain some of the not replicated findings in literature. Moreover, previous studies which investigated only the long/short polymorphism should be reconsidered.

SNPs occur, on average, about less than 1.000 bases. Since there are about 3 billion chemical base pairs that make up human DNA and its 20.000 – 25.000 genes, about 3 - 5 million of SNPs might be expected. This is consistent with the recent phase II Hap Map project findings [10]. The analysis of such a number of variations is feasible from a long time: actual techniques (Illumina and Affymetrix) permit the analysis of 500,000 or more SNPs in a single test with accessible costs, and lists of TagSNPs which can cover the complete gene sequence are easily retrievable from public databases (http://www.hapmap.org/downloads/index.html.en) (http://www.ncbi.nlm.nih.gov/projects/mapview/map_search.cgi?taxid=9606). 

Moreover, online free software is available which identify is a list of significant SNPs to cover a gene’s common variations (http://www.broad.mit.edu/mpg/haploview/down-load.php) ((http://marketing.appliedbiosystems.com/mk/get/SNPB_LANDING?_A=22924&_D=18392&_V=0).).

As a conclusion, a more complete analysis of genes’ variations does not represent a novelty in actual knowledge, and its rationality as a methodological strategy is expected to be self – established. Nonetheless, we report here that it is a poorly considered methodological point which could be easily fixed using free internet resources and laboratory extra work, likely affordable in studies able to perform genetic and clinic assessments of hundreds of patients. There is some evidence that every day genetic clinic use will be a cost effective or even cost-saving approach in general clinical practice [31]: in order to hasten this process, a complete coverage of single gene polymorphisms is probably needed.

## Figures and Tables

**Table 1:** Examples of Investigated/Known Variations Ratio

Author	Sample	Gene (s)	Variation (Investigated / Known)	Number of Tag SNPs	Number of LD Blocks	Main Association Result
[32]	1447	CREB 1	7 / 173	18	4	Positive association in men (suicide)
[33]	854	HTR2C; HTR1A	6 / 1100 (HTR2C); 3 / 16 (HTR1A)	18 (HTR2C) No haplotypes (HTR1A)	4 (HTR2C) No haplotypes (HTR1A)	No correlation
[34]	1913	SLC6A4	1 / 171	14	1	No correlation (MDD, positive correlation if stress associated; alcohol dependence)
[35]	1435 (meta analysis)	SLC6A4	1 / 171	14	1	Positive correlation (MDD, AD treatment)
[36]	1914	SLC6A4	1 / 171	14	1	No correlation (AD treatment)
[37]	1648 (meta analysis)	MTHFR	2 / 147	9	1	Positive correlation (depression, anxiety, psychosis)
[38]	9032 (meta analysis)	SLC6A4	1 / 171	14	1	Positive correlation (sucide; p = 0.0068)
[39]	3000 (meta analysis)	SLC6A4	1 / 171	14	1	Positive correlation (OCD)
[40]	195	GNbeta3	1 / 39	3	No haplotypes	Positive correlation (self mutilation)
[41]	222	HTR1A	2 / 16	No haplotypes	No haplotypes	Positive correlation (AD treatment, females)
[42]	4175 (298 MDD)	SLC6A4	1 / 171	14	1	No correlation
[43]	258	SLC6A4	1 / 171	14	1	Positive correlation (suicide)
[44]	196	DRD4	1 / 106	No haplotypes	No haplotypes	Positive correlation (neuroticism)
[45]	450	GLO1	1 / 152	11	2	Positive correlation (panic without agoraphobia)
[46]	937	GAL	4 / 30	3	1	Positive correlation (alcoholism)
[47]	755	DTNBP1	2 / 448	26	2	Positive correlation (negative symptoms of schizophrenia)
[48]	2376	BDNF	3 / 214	5	1	Positive correlation (MDD)
[49]	178	CLOCK	1 / 523	24	5	Positive correlation (AD treatment insomnia)
[50]	273	ACE; ATR1	(1 + 1) / (259 + 422 )	11 (ACE) 18 (ATR1)	4 (ACE) 2 (ATR1)	Positive correlation (AD treatment)
[51]	1512	COMT	1 / 293	14	3	Positive correlation (MDD)
[52]	753	GPR50	3 / 29	No haplotypes	No haplotypes	Positive correlation (DB)
[53]	1005	SLC6A4	1 / 171	14	1	Positive correlation (gene environment influence)
[54]	295	SLC6A4	1 / 171	14	1	No correlation (OCD)
[55]	159	GABBR1	5 / 204	21	6	Positive correlation (OCD)
[56]	287	BDNF	2 / 214	5	1	No correlation
[57]	273	21 candidate genes	90 polymorphisms (average = 3.5)	Different genes	Different genes	Positive association (PD)
[58]	230	NET	3 / 263	25	4	Positive correlation (PD without agoraphobia)
[59]	373	SCL6A4; MAOA; TPH1; HTR1B	2 / 171 (SLC6A4); 1 / 165 (MAOA); 1 / 81 (TPH1); 1 / 25 (HTR1B)	14 (SLC6A4) 8 (MAOA) 2 (TPH1) 4 (HTR1B)	1 (SLC6A4) 1 (MAOA) None (TPH1) 1 (HTR1B)	No correlation
[60]	65 families	PIP5K2A	15 / 742	53	12	Positive association
[61]	433	FZD3	2 / 280	4	1	No correlation
[62]	944	BDNF	2 / 214	5	1	Positive association
[63]	896	SYN3	1 / 2742	257	45	No correlation
[64]	1153	RGS4	4 / 36	6	1	No correlation

Gene names are the officials ones according to NCBI database. To identify the Tag SNPs Haploviewer tagger program was used with default settings, CEU population was selected.

**Table 2:** Papers Published in 2006–7 that Reported Positive Association Results for Intronic SNPs

Author	Disorder	Chromosome	Gene	Variation (Intron)	Distance from the Nearest Exon
[65]	Psychosis	22q13.33	MLC1	rs2235349; rs2076137	~ 100 bp and 65 bp
[66]	Psychosis	18p11.3	TGIF	D18S63	~ 9.2 kbp
[67]	PTSD	Xp11.23	MAO-B	rs1799836	~ 30 bp
[68]	BD	14q22.3	OTX2	rs28757218	~ 20 bp
[69]	Personality	6p12.3	TFAP2B	VNTR (intron 2)	~ 100 bp
[70]	Antipsychotic treatment	7q36.1	NOS3	VNTR (intron 4)	~ 200 bp
[71]	Lithium treatment response	17q11.2	SLC6A4	VNTR (intron2)	~ 100 bp
[72]	Antidepressant response	5p15.33	DAT1	VNTR (intron 8)	~ 200 bp
[73]	Antidepressant response	Xp11.23	MAO-B	rs1799836	~ 30 bp
[74]	Eating disorder spectrum	3p25.3	GHRL	rs35680	~ 800 bp
[75]	Personality	19q13.42	PRKCG	rs402691	1.2 kbp
[76]	Psychosis	22q11.21	COMT	rs737865; rs737864	~ 700 kb
[77]	Depressive disorder	17q11.2	SLC6A4	rs25531	~ 1.5 kbp
[78]	Psychosis	6p21.31	FKBP5	17081296	~ 2.5 kbp
[79]	Antipsychotic response	15q24.1	CYP1A2	rs2472304	~ 40 bp
[80]	BD	8p21	VMAT1	rs2279709	~ 220 bp
[81]	Psychosis	8p12	NRG1	rs6150532	~ 700 bp
[82]	BD	21q22.3	TRPM2	rs1618355	~ 15 bp
[83]	Suicide	11p15.1	TPH-1	rs684302; rs211105; rs1800532; rs7933505	~ 250 to 2000 bp
[78]	Psychosis	6p21.31	FKBP5	rs1360780	~ 1.5 kbp
[44]	Personality	7p15.1	CRHR2	rs2267717	~4.5 kbp
[84]	Suicide	11p15.1	TPH	Intron 7 SNPs	< 750 bp
[85]	Depressive disorder	11q23.2	HTR3A and HTR3B	rs2276307; rs2276308; rs3782025; rs2276302	From 60 to 150 bp

Gene names are the officials ones according to NCBI database.
